# Selective sweep analysis reveals extensive parallel selection traits between large white and Duroc pigs

**DOI:** 10.1111/eva.13085

**Published:** 2020-08-28

**Authors:** Saixian Zhang, Kaili Zhang, Xia Peng, Huiwen Zhan, Jiahui Lu, Shengsong Xie, Shuhong Zhao, Xinyun Li, Yunlong Ma

**Affiliations:** ^1^ Key Laboratory of Agricultural Animal Genetics, Breeding, and Reproduction of the Ministry of Education & Key Laboratory of Swine Genetics and Breeding of the Ministry of Agriculture Huazhong Agricultural University Wuhan China

**Keywords:** parallel selection signatures, pig, quantitative traits

## Abstract

In the process of pig genetic improvement, different commercial breeds have been bred for the same purpose, improving meat production. Most of the economic traits, such as growth and fertility, have been selected similarly despite the discrepant selection pressure, which is known as parallel selection. Here, 28 whole‐genome sequencing data of Danish large white pigs with an approximately 25‐fold depth each were generated, resulting in about 12 million high‐quality SNPs for each individual. Combined with the sequencing data of 27 Duroc and 23 European wild boars, we investigated the parallel selection of Danish large white and Duroc pigs using two complementary methods, Fst and iHS. In total, 67 candidate regions were identified as the signatures of parallel selection. The genes in candidate regions of parallel selection were mainly associated with sensory perception, growth rate, and body size. Further functional annotation suggested that the striking consistency of the terms may be caused by the polygenetic basis of quantitative traits, and revealing the complex genetic basis of parallel selection. Besides, some unique terms were enriched in population‐specific selection regions, such as the limb development‐related terms enriched in Duroc‐specific selection regions, suggesting unique selections of breed‐specific selected traits. These results will help us better understand the parallel selection process of different breeds. Moreover, we identified several potential causal SNPs that may contribute to the pig genetic breeding process.

## 1 INTRODUCTION

The domestication of animals is very important in human history, which has led to the transformation of human life from hunting and gathering to an agricultural lifestyle. As one of the most important livestock, pigs were domesticated in multiple locations approximately 9,000 years ago (Giuffra et al., 2000; Larson et al., 2005), leading to a series of changes involving behavior, morphology, and physiology (Groenen, 2016; Larson & Burger, 2013; Ramos‐Onsins, Burgos‐Paz, Manunza, & Amills, 2014). And then, artificial selection has been conducted to improve agriculturally important traits, which not only further results in population diversity, but also makes a few similar characters of the populations with the same breeding objectives, such as growth rate and dietary habits (Rubin et al., 2012). Therefore, it is important to understand how domestication and artificial selection have shaped pig genome, which can provide valuable insights for further improvement of economic traits in pigs.

When a favorable mutation emerges, its frequency will increase rapidly due to natural or artificial selection and this process is called selective sweep (Smith & Haigh, 1974). In general, selective sweep can lead to long haplotypes, high‐frequency derived alleles, and highly differentiated alleles (Grossman et al., 2010). With the implementation of high‐throughput genotyping techniques, identifying selective sweeps at the genome level has become possible. For example, Rubin et al. identified three genes (*NR6A1*, *PLAG1,* and *LCORL*) that contribute to the body length of European domesticated pigs (Rubin et al., 2012). Wang et al. (2015) revealed one synonymous substitution in *ESR1,* which may influence litter size and two genes related to coat color in Tongcheng pigs. Ma et al. (2018) revealed strong signatures of selection in Duroc that can affect lean muscle mass, fertility, and immunization. However, the selection signatures shaped by similar breeding direction among commercial pig breeds in recent years have not been investigated in depth.

Large white and Duroc, two famous commercial pig breeds, are widely used in pig industry. Although there are different breeding objectives of the two breeds, both of them have demonstrated similar superior performance in many traits such as growth rate and meat quality due to the long‐term intense artificial selection. From the perspective of population genetics, the genomic patterns shaped by similar selection directions between populations could be termed as parallel selection signatures. So far, the traits underlying parallel selection have been reported in several species, such as herring (Lamichhaney et al., 2017) and caribou (Horn et al., 2018). In pigs, Frantz et al. (2015) reported a parallel selection in *PLAG1* region between Asian and European domestic pigs. However, a further investigation of the parallel selection in pigs has not been reported, especially in the different commercial breeds under intense artificial selection. Thus, we conducted an analysis of the parallel selection between Danish large white (DLW) and Duroc (DU) in this study.

To reveal the genetic basis of parallel selection traits between DLW and DU, 28 and 27 resequencing data of DLW and DU were used in this study, and 23 resequencing data of European wild boar (EWB) were used as reference population. In general, it will be promising to get a reliable result using at least 15 samples according to the previous study (Ma et al., 2015). By combining Fst and iHS methods, we identified 67 promising parallel selective signatures between DLW and DU. With the annotations of these selection regions by GO and MGI database, we found that the annotated terms of selection signatures imply a complex genetic basis of the parallel selection traits. Moreover, several putative causative mutations were identified that may influence the traits involving immune, fertilization, and embryo development, which can contribute to further pig breeding processes.

## 2 MATERIALS AND METHODS

### 2.1 Sample collection, genome sequencing, and quality control

The genomic DNA was extracted from ear tissues of 28 Danish large white pigs and four Duroc pigs with a standard phenol–chloroform method. All research involving animals was conducted under protocols (No. 5 Proclaim of the Standing Committee of Hubei People's Congress) approved by the Standing Committee of Hubei People's Congress and the Ethics Committee of Huazhong Agricultural University in China. All experiments were performed in accordance with approved relevant guidelines and regulations. For each sample, we constructed a paired‐end sequencing library with a 350 bp insert size. The libraries were then sequenced with 2 × 150 bp paired‐end reads on Illumina HiSeq X Ten platform at the BGI‐Huada Genomics Institute in Shenzhen. In addition, we also downloaded the whole‐genome resequencing data of 46 *Sus scrofa* individuals (23 Duroc pigs and 23 European wild boars) from the EMBL‐EBI database (https://www.ebi.ac.uk/) (Bosse et al., 2014, Kim et al., 2015, Ramirez et al., 2015). To reduce artificial bias in sequencing process, Trimmomatic v0.36 (Bolger, Lohse, & Usadel, 2014) and NGS‐QC Toolkit v2.3.3 (Patel & Jain, 2012) were used to remove the reads with following criteria: (a) reads with more than 10 bp aligned to adapter with up to 10% mismatches; (b) reads with up to 10% unidentified nucleotides (N); (c) reads with more than 50% bases having a Phred quality less than 5; and (d) duplicate reads generated by PCR amplification in library construction process. Detailed information of samples was shown in Table S1.

### 2.2 Read mapping, variant calling, and annotation

The cleaned reads were mapped to the pig reference genome (Sscrofa11.1) with the Burrows–Wheeler Aligner software v0.7.17 (Li & Durbin, 2009), employing the “mem” algorithm with default parameters. After alignment, we used SAMtools v1.3.1 (Li et al., 2009) to convert the SA coordinates to the best alignments in BAM format. The “CreateSequenceDictionary,” “SortSam,” and “MarkDuplicates” of Picard software v1.119 (https://broadinstitute.github.io/picard/) were used to index, sort, and remove potential PCR duplicates separately. Using SAMtools, we created index files for the reference genome and bam files. We then used the “HaplotypeCaller,” “SelectVariants,” and “VariantFiltration” of GATK v3.8 (McKenna et al., 2010) with default parameters to call SNPs. Furthermore, the SNPs called by SAMtools “mpileup” and BCFtools v1.3.1 (Heng Li et al., 2009) were used to correct the results of GATK. Finally, high‐quality SNPs with (a) average coverage depth ≥5, (b) RMS mapping quality ≥20, (c) the distance of adjacent SNPs ≥5 bp, and (d) the missing ratio of samples <10% were kept for further analysis. All the filtered SNPs were functionally annotated with ANNOVAR (Wang, Li, & Hakonarson, 2010) and the Ensembl pig gene set version 94 (Sscrofa11.1.94).

### 2.3 Identification of selection signatures

In this study, Weir and Cockerham's Fst (Weir & Cockerham, 1984) and integrated haplotype score (iHS) (Voight, Kudaravalli, Wen, & Pritchard, 2006) were used to detect selection signatures. We calculated the Fst values using VCFtools v0.1.15 (Danecek et al., 2011), and the Fst scores of the three comparisons were represented as *F*_ST|DLWvsEWB_, *F*_ST|DUvsEWB_, and *F*_ST|DLWvsDU_ in this research, respectively. For iHS test, we first phased the data set using Beagle version 5.0 (Browning & Browning, 2007). Then, scores were calculated for each SNP of the phased data and standardized within each of 100 bins of allele frequency using selscan software v1.2.0a (Szpiech & Hernandez, 2014) and represented as iHS_DLW_, iHS_DU,_ and iHS_EWB_, respectively. Finally, the absolute iHS scores were averaged into small windows for further analysis. Both the methods were detected with a sliding window of 50 kb with a step size of 25 kb, and the windows with <10 SNPs were discarded. The empirical P‐values were generated by genome‐wide ranking of all comparisons, and the windows with top 1% values were considered as candidate selection regions. The identified regions were then extended by 200 kb to each side which was determined by the linkage disequilibrium decay. Finally, we merged the continuous windows within each comparison and populations with BEDTools software v2.26.0 (Quinlan & Hall, 2010).

#### 2.3.1 Detecting artificial selection signatures

From the perspective of population genetics, Fst method is more suitable for detecting selection signatures that occurred in further time (Cadzow et al., 2014), while iHS method is more suited for detecting recent selection (Voight et al., 2006). Taking EWB as reference, we thus defined the artificial selection of DLW by merging the regions detected by *F*_ST|DLWvsEWB_ and iHS_DLW_, and then excluded the regions which were identified as outliers by iHS_EWB_. The same process was used for DU, and the *F*_ST|DUvsEWB_ and iHS_DU_ were merged and followed by excluding the regions identified by iHS_EWB_. The processes could be represented as (*F*_ST|DLWvsEWB_ ∪ iHS_DLW_) − iHS_EWB_ and (*F*_ST|DUvsEWB_ ∪ iHS_DU_) − iHS_EWB_, respectively.

#### 2.3.2 Detecting parallel selection signatures

To reveal the potential parallel selection signatures, genomic regions identified by Fst and iHS were firstly processed to extract overlapped regions, respectively. And then, the overlapped regions detected by Fst and iHS were merged followed by excluding regions identified by different comparisons of Fst.

More specifically, for parallel selection, we first extracted the overlapped regions of *F*_ST|DLWvsEWB_ and *F*_ST|DUvsEWB;_ meanwhile, the overlapped regions of iHS_DLW_ and iHS_DU_ were extracted and followed by excluding the regions identified by iHS_EWB_. We then merged the regions obtained from Fst and iHS methods and excluded regions of *F*_ST|DLWvsDU_. This process was represented as (*F*_ST|DLWvsEWB_ ∩ *F*_ST|DUvsEWB_) ∪ (iHS_DLW_ ∩ iHS_DU_ − iHS_EWB_) − *F*_ST|DLWvsDU_.

In addition, we noted that most selection signatures were only detected either in large white or in Duroc pigs in comparison with parallel selection signatures. Therefore, we also have conducted in‐depth investigations of such genomic selection regions. The similar processes were separately performed to identified specific selection regions in DLW and DU. The population‐specific signatures could be represented as (*F*_ST|DLWvsEWB_ ∩ *F*_ST|DLWvsDU_) ∪ (iHS_DLW_ − iHS_DU_ − iHS_EWB_) − *F*_ST|DUvsEWB_ and (*F*_ST|DUvsEWB_ ∩ *F*_ST|DLWvsDU_) ∪ (iHS_DU_ − iHS_DLW_ − iHS_EWB_) − *F*_ST|DLWvsEWB_, respectively. The parallel selection, DLW‐specific selection, and DU‐specific selection were indicated as PS, DLW.sp, and DU.sp, respectively.

### 2.4 Quantitative trait loci mapping

The pig quantitative trait loci (QTL) database (Hu, Park, Wu, & Reecy, 2012) was searched to find the known QTLs that overlapped with each detected selection region. We firstly downloaded the pig QTLs from the database and removed QTLs with uncertain genomic locations or length more than 1 Mb, resulting in 21,952 QTLs. Most of the resource populations used in the researches involved in these remaining QTLs include the large white and Duroc pig. Therefore, we did not further filtration by breeds due to the complex background of these QTLs. After the comparison of QTLs and candidate regions, only the regions with more than two QTLs that have consistent associated traits were remained. These regions were further classified into five groups defined in the QTL database: meat and carcass, health, exterior, production, and reproduction traits.

### 2.5 Annotation of selected genes

Genes located in selection regions were identified through the BioMart of Ensembl (Durinck et al., 2005). Enrichment analysis of Gene Ontology (GO) was performed using Panther web server (Thomas et al., 2006), and the terms with *p*‐value smaller than .05 (Fisher's extract test) and more than one gene were retained. For better understanding the phylotypes that the candidate genes may involve in, we conducted an overrepresentation analysis using MGI database (Richardson & Bult, 2015). The conversion of gene ID between pig and mouse was performed using BioMart.

### 2.6 Identification of putative functional SNPs

To identify putative functional SNPs, we first extracted the variants annotated as nonsynonymous located within the selective regions. And then, allele frequency difference (ΔAF) for each variant was calculated and the variants with absolute ΔAF ≥0.8 were remained for further analysis. Besides, genes without gene symbols were excluded due to the little knowledge about them. The ΔAF was defined as (AF_DLW_ + AF_DU_)/2 − AF_EWB_, AF_DLW_ − (AF_DU_ + AF_EWB_)/2 and AF_DU_ − (AF_DLW_ + AF_EWB_)/2 for parallel selection, and specific selection of DLW and DU, respectively.

We further predicted the effect of the remained SNPs on protein function using SIFT4G, a faster version of SIFT (Sorting Intolerant From Tolerant) software (Kumar, Henikoff, & Ng, 2009; Vaser, Adusumalli, Leng, Sikic, & Ng, 2016). Before the prediction, we built a custom database used by SIFT4G for Sscrofa11.1. SIFT4G outputs whether a SNP is deleterious or tolerate for each SNP, and a score is also assigned. The scores of SIFT4G range from 0 to 1, and SNPs are predicted to be deleterious if the score ≤0.05 and tolerate if the score >0.05.

## 3 RESULTS

### 3.1 Sequencing, SNP calling, and annotation

Whole‐genome sequencing of 28 Danish large white pigs was performed, resulting in ~1,603.98 Gb in total with ~23.52× depth per individual (Table S1), which enabled us to obtain nearly complete genetic variants and identify a genome‐wide set of candidate regions for artificial selection in Danish large white pigs. A total of 5.35 billion cleaned paired‐end reads were generated, and 5.32 billion (99.44%) reads were successfully aligned to Sus scrofa 11.1 reference genome with the Burrows–Wheeler Alignment (BWA). Consequently, the average sequencing depth was 23.52× and approximately 99.31% of the reference genome was covered by reads for each sample (Table S1), allowing us to call variants with high confidence.

After applying stringent quality control criteria, a total of 10.81 million SNPs with high quality were identified and shown in Table 1. To accurately detect signatures left by select, we downloaded 23 Duroc and 23 European wild boar (EWB) from publicly available resequencing database. Combining with resequencing data of four Duroc pigs previously performed in our laboratory, we detected 10.39 and 10.15 million SNPs in Duroc and EWB, respectively (Table 1), following the same pipeline and quality control criteria with DLW data. Overall, 1,080,927 million SNPs were detected by our data and then compared to the pig dbSNP database (Build 150; Figure S1). Over 80% of the variants in our data set were found in the dbSNP database, demonstrating the high quality and reliability of our SNP data set, and the novel SNPs substantially expand the catalog of porcine genetic variants.

**TABLE 1:** Summary of sequencing data and SNPs in DLW, DU, and EWB

Fields	DLW	DU	EWB	Overall
Sample counts	28	27	23	78
Average depth (X)	23.52	12.43	9.67	15.21
Average genome coverage (%)	99.31	96.56	86.77	94.21
Average mapping rate (%)	99.44	99.67	99.77	99.63
High‐quality bases (Gb)	1603.98	817.35	541.45	2,962.79
Q20 (%)	95.48	97.36	97.89	96.91
Q30 (%)	89.21	91.18	91.50	90.63
SNP categories				
Number of total SNP	10,635,485	7,918,935	8,582,578	10,809,276
Upstream	62,281	41,584	45,258	63,487
UTR5	19,118	11,141	11,555	19,373
Stop gain	278	186	205	279
Stop loss	58	38	40	58
Exonic				
Synonymous	45,246	27,575	28,412	45,553
Nonsynonymous	24,919	16,006	16,787	25,200
Unknown	37	77	126	135
Splicing	376	240	276	382
Intronic	4,112,359	3,011,754	3,246,827	4,178,406
UTR3	99,978	70,233	75,627	101,392
UTR5/UTR3	534	315	316	538
Downstream	67,917	47,606	51,334	69,160
Upstream/downstream	1,863	1,168	1,242	1,885
Intergenic	6,200,521	4,691,012	5,104,573	6,303,428

Abbreviations: DLW, Danish large white; DU, Duroc; EWB, European wild boar.

The identified SNPs were then further annotated using ANNOVAR software. Consistent with previous studies (Ai et al., 2015; Li et al., 2014; Zhao et al., 2018), most SNPs (58.35%) were located in intergenic regions, followed by intronic regions (38.68%; Table 1). Besides, 0.28 M (0.68%) SNPs were identified in coding regions, of which 101,153 nonsynonymous variants (99,683 missense, 1,256 stop gain, and 214 stop loss) were detected in 15,100 genes, which might be associated with changed traits in pigs. We then performed GO analysis based on the 1,000 genes which contain the highest number of nonsynonymous variants. The mainly over‐represented terms were olfactory‐related categories (Table S2), which was consistent with previous studies (Fu et al., 2016; Li et al., 2014). As one of the largest gene families in pig (Groenen et al., 2012), olfactory receptor genes are also belong to the most rapidly evolving genes (Paudel et al., 2013), which could thus lead to the intense selection.

### 3.2 Genome‐wide artificial selection signatures

Based on the high‐quality SNPs, we then used Fst and iHS methods to detect genome‐wide selection signatures of DLW, DU, and EWB. For both methods, a sliding window of 50 kb with a step of 25 kb was used, and windows with more than 10 SNPs were remained for further analysis. As result, we got 86,959, 86,966, and 86,251 windows of Fst values of *F*_ST|DLWvsEWB_, *F*_ST|DUvsEWB_, and *F*_ST|DLWvsDU_, and 90,622 windows of iHS values of these three populations. We then used an empirical P‐value method by ranking Fst and iHS scores of all windows in each population and defined the windows with *p*‐value <.01 as selection regions. By combining adjacent regions, we then obtained the candidate selection regions of each method in each population (Table S3).

**FIGURE 1:**
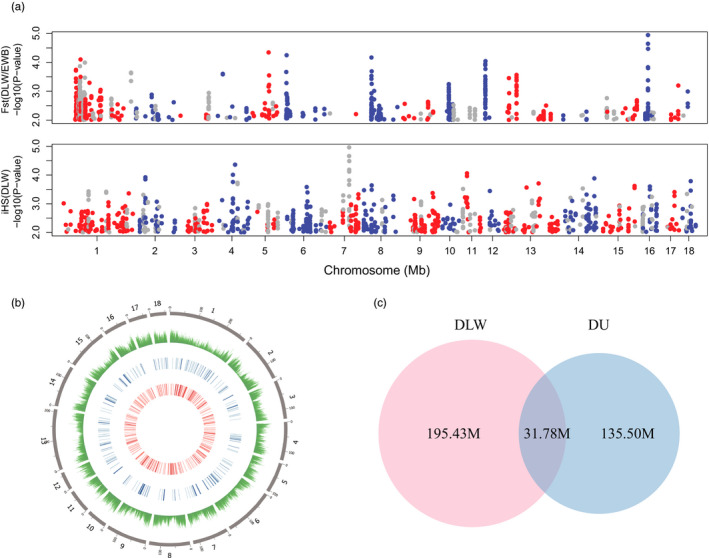
Candidate selection regions of DLW and DU. (a) Candidate selection regions of DLW detected by two statistics (Fst and iHS) are plotted across the genome. Red and blue dots represent the regions that are not identified as outliers in EWB with iHS method, and gray dots represent the regions that are identified as outliers in EWB with iHS method. (b) Circos plot of distribution of selection regions of DLW and DU in genome. The circles from outside to inside illustrate SNP density and selection regions of DU and DLW, respectively. (c) Venn diagram shows overlapped size of selection regions between DLW and DU. DLW, Danish large white; DU, Duroc; EWB, European wild boar

To identify artificial selection in DLW, we merged the regions detected by iHS in DLW and the Fst of comparison between DLW and EWB. And the same process was conducted for DU. In total, we obtained 392 and 232 candidate selection regions in DLW and DU, respectively (Figure 1a, Figure S2, and Table S4). By comparing the candidate selection regions identified in DLW and DU, we found that a total of 31.78 M regions were shared by DLW (13.99%) and DU (19.00%; Figure 1b,c), which suggested the existence of parallel selection between DLW and DU.

### 3.3 Genes, GO terms, and QTLs suggesting parallel selection in the progress of artificial selection

To further investigate the relation between the selection regions of DLW and DU, we performed GO analysis based on the genes detected in DLW and DU, respectively. As shown in Tables S5–S8, the most over‐represented terms in both breeds were related to olfaction which has been reported in several studies (Amaral et al., 2011; Groenen et al., 2012; Li et al., 2013). Pig has one of the most olfactory receptor (OR) repertoires (Nguyen et al., 2012), and *OR* genes are also the most rapidly evolving genes in pigs (Paudel et al., 2015). The reason of the rapid evolution of *OR* genes could be the need for adapting to new environment; moreover, *OR* genes might also influence mate choice (Paudel et al., 2013). These findings can further support the hypothesis of selection and rapid evolution for *OR* genes during domestication. Besides the terms involved in olfaction, some other trait‐related terms were also shared between two populations such as embryonic development, nerve system, and growth.

Quantitative trait loci mapping analysis was then performed. In total, 685 (76 terms) and 740 (72 terms) QTLs from pig QTL database were mapped to the selection regions of DLW (Table S9) and DU (Table S10), respectively. Among these QTLs, 42 terms were shared in both populations such as “Daily feed intake,” “Lean meat percentage,” and “CD8‐positive leukocyte percentage.” The shared GO and QTL terms between DLW and DU are highly related to some commercial traits such as growth, fertility, and immune which suggested the parallel selection under the domestication.

### 3.4 Evidence of parallel selection, 67 promising signatures with long‐range haplotype homozygosity

Ideally, the signatures of parallel selection will display the similar genomic characters when a genomic region has been shaped by similar artificial selection in different populations. In this analysis, we searched for all selection signatures that overlapped with each other between large white and Duroc pigs. In total, 67 genomic regions were simultaneously detected in the large white and Duroc pigs, covering 1.03% of the genome and containing 240 genes.

As shown in Table 2, many genes identified here were also detected in previous studies, such as *NR6A1* and *PLAG1* (Rubin et al., 2012). *NR6A1* (nuclear receptor subfamily six group A member 1) has been reported to be associated with the vertebral number (Mikawa et al., 2007). The vertebral number is important in pig breeding for its effect for body size and meat production (Yang, Ren, Zhang, & Huang, 2009). Compared with wild boars and indigenous breeds which have 19 vertebrae, the number of vertebrae of Western commercial breeds has increased to 21–23 due to the selective breeding for enlarged body size (Yang et al., 2009). And *PLAG1* (PLAG1 zinc finger) is mainly involved in height (Gudbjartsson et al., 2008; Karim et al., 2011), which can also influence the body size in pigs.

**TABLE 2:** Some detected genes of artificial selection

Chr.	Pos	Gene	P‐value	Selected population	Gene function
1	89994480–90084765	*MYO6*	0.001	DLW	Hearing/vestibular/ear system (Avraham et al., 1995)
1	265322015–265570887	*NR6A1*	<0.001	DLW, DU	Vertebral number (Mikawa et al., 2007)
2	134986599–134994365	*IL−4*	0.001	DU	Immunity (McLeod et al.,^2015^)
2	136494395–136522681	*PPP2CA*	0.004	DU	Embryonic development (Gu et al., 2012)
					Fertility (Pan et al., 2015)
4	75646592–75694365	*PLAG1*	0.009	DLW, DU	Body size (Gudbjartsson et al., 2008)
8	40977604–41018506	*PDGFRA*	0.001	DLW	Intramuscular fat (Sun et al., 2017)
					Fertility (Schmahl et al.,^2008^)
9	139301586–139475037	*EGFR*	0.004	DLW	Embryonic development (Steffl et al., 2005)
					Fertility (Li et al., 2008)
12	25197257–25242918	*IGF2BP1*	0.005	DLW, DU	Growth, intestinal development (Hansen et al.,^2004^)
13	51178185–51422093	*MITF*	<0.001	DU	Pigmentation (Kawakami et al., 2017)
15	48053367–48106860	*FGFR1*	0.007	DLW	Uterine development (Welter et al., 2004)
					Hearing (Pirvola et al., 2002)
					Embryonic development (Yamaguchi et al., 1994)
					Limb development (Verheyden et al., 2005)
18	2545718–2558517	*SHH*	0.022	DLW, DU	Embryonic development (Nguyen et al., 2009)
18	45393945–45402617	*HOXA10*	0.003	DU	Fertility (Du et al. 2016)

Abbreviations: DLW, Danish large white; DU, Duroc.

Quantitative trait loci were then mapped to parallel selection regions, and a total of 26 QTLs were found (Table S11 and S12). Among the five catalogs, the number of QTLs associated with “Meat and Carcass” was 16, accounting for 61.5% of the total QTLs. As important economic traits, “Meat and Carcass”‐associated traits, such as body size and intramuscular fat content, have undergone intense artificial selection. In addition, “coping behavior”‐related QTLs were relatively highly enriched. Coping behavior is defined as the behavior of pig response to aversive situations (Wechsler, 1995). Aversive environment may lead to a fitness if animals cannot cope with it, thus result in selection on animal in this situation (Broom, 1991). For pigs, intense feeding pattern has been greatly developed in recent years, thus may have led to the selection of “coping behavior” (Table S12).

**FIGURE 2:**
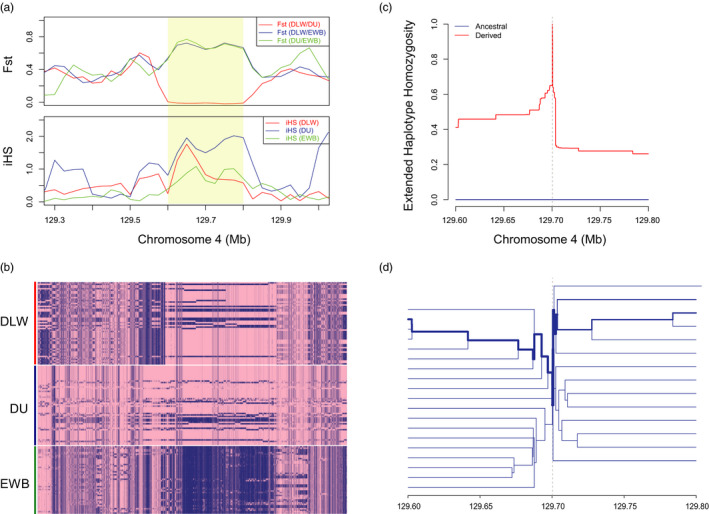
A parallel selective sweep region in chromosome 4. (a) Plot of statistics (Fst and iHS) over an approximately 700‐kb region in chromosome 4, including population differentiation (Fst) between each pair of DLW, DU, and EWB, and iHS of each population. (b) Heatmap of haplotype of the region among the three populations (DLW, DU, and EWB). The allele which is consistent with reference genome is indicated in pink and another allele in blue. (c) The EHH plot shows a long‐conserved haplotype in this region. (d) Bifurcation diagrams of the region

In addition, we also found that most of the 67 genomic regions displayed a similar long‐range haplotype homozygosity between large white and Duroc pigs, which can provide an evidence for parallel selection. As the region is shown in Figure 2a, Fst statistics *F*_ST|DLWvsEWB_ and *F*_ST|DUvsEWB_ showed similar and high values, while *F*_ST|DLWvsDU_ is on a very low level, and iHS scores of DLW and DU in this region are distinctly higher than EWB, suggesting that both DLW and DU are under a strong parallel selection in this region. Moreover, the haplotypes of all three breeds in this region (Figure 2b) show the same situation as Fst and iHS, DLW, and DU have similar haplotype patterns, while EWB has a distinctive pattern, indicating a similar strong selection for both DLW and DU in this region. Besides, we constructed the extended haplotype homozygosity in this region using rehh package (Gautier, Klassmann, & Vitalis, 2017). As shown in Figure 2c,d, a prominent haplotype was presented, indicating a strong parallel selection between DLW and DU. This region contains three genes, *CLCA1* (chloride channel accessory 1), *CLCA2* (chloride channel accessory 1), and *ODF2L* (outer dense fiber of sperm tails 2 like). *CLCA1* and *CLCA2* are both belong to the calcium‐sensitive chloride conductance protein family and related to several diseases (Gruber et al., 1998; Ponsard, Seltzer, Perret, Tournier, & Middendorp, 2007; Ritzka et al., 2004), and *ODF2L* can be important in embryogenesis (Nigg & Raff, 2009).

Based on the identified 240 genes, we then performed a gene ontology enrichment analysis using Panther (Table S13). By using Fisher's extract test, 225 gene ontologies were found to be significantly enriched (*p*‐value <.05) and the top 20 terms are shown in Figure S3a. The most significant term was “sensory perception,” which was consistent with before. Besides, some nerve and immune‐related terms were highly over‐represented in our result. The nervous system‐related terms have been reported in several studies (Frantz et al., 2015; Moon et al., 2015; Wang et al., 2015), which could be involved in behavior and tameness that have been under selection during the long‐term domesticate process. Similar to sensory perception, immunity‐related genes were among the genes with the most rapidly evolving speed (Paudel et al., 2013).

To better understand the function of candidate genes, a phenotype enrichment analysis was performed using MGI database (http://www.informatics.jax.org) which allows us to link the genes to traits. As shown in Figure S3b, the most significant term was “cellular process,” followed by “growth/size/body region,” “mortality/aging,” and “reproduction system.” The result was consistent with the fact that DLW and DU have better performance than wild boar in “reproduction” and “growth,” and indicated the genes in these terms may be responsible for the corresponding phenotypic changes in domestication pigs.

### 3.5 Population‐specific selection signatures reveal a polygenic basis of parallel selection traits

In addition to the overlapped selection signatures, we also identified some population‐specific selection signatures of large white and Duroc pigs, respectively. Here, the population‐specific selection signatures were defined as the genomic regions that artificial selection was only detected in one of two domestic pig breeds, either large white or Duroc pigs. Totally, 268 genomic regions, covering 6.13% of the genome and containing 1708 genes, were identified in large white pigs. Similarly, 70 genomic regions, covering 1.47% of the genome and containing 431 genes, were identified in Duroc pigs.

Five hundred and seventy‐six (115 terms) and 247 (33 terms) QTLs were found mapped to specific selection regions of DLW and DU, respectively (Tables S11, S14, and S15). Among them, most QTLs that were identified separately in two breeds are associated with the same traits, such as lean meat percentage, body length, coping behavior, litter weight, and intramuscular fat content. The same 17 terms of these QTLs implied that these traits were subjected to parallel selection during the artificial selection process of the two breeds, and the genetic basis of these parallel selection traits is polygenic. Taken intramuscular fat content as an example, 2, 7, and 2 QTLs have been found separately in the parallel selection region in chromosome 9, DU‐specific selection region in chromosome 12 and DLW‐specific selection region in chromosome 9. This may be an example of parallel selection traits that are controlled by polygenic basis.

**FIGURE 3:**
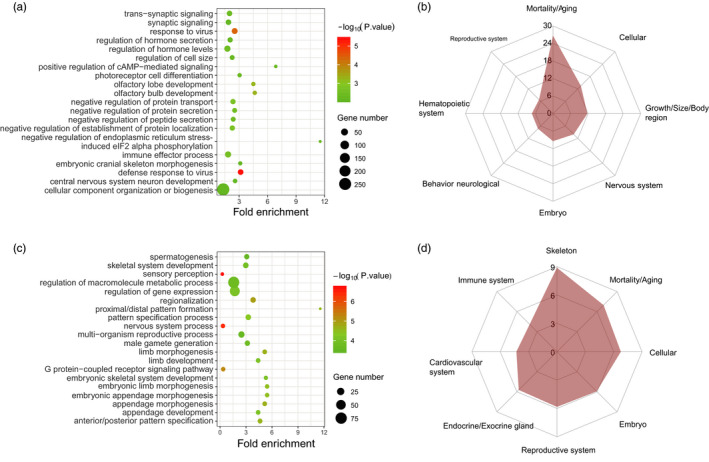
Annotation of the genes detected by DLW‐ and DU‐specific selection, respectively. (a) Significantly enriched GO terms (top 20) of DLW‐specific selection. (b) Significantly enriched MGI terms (top 8) of DLW‐specific selection. The y‐axis in MGI plot indicates −log10(*p*‐value). (c) Significantly enriched GO terms (top 20) of DU‐specific selection. (d) Significantly enriched MGI terms (top 8) of DU‐specific selection. The y‐axis in MGI plot indicates −log10(*p*‐value)

To further investigate parallel selection traits from the potential function of population‐specific selection signatures, the genes overlapped with the selection regions in each breed were annotated with GO and MGI databases, respectively. As shown in Figure 3a,c, the top 20 terms of large white and Duroc pigs were chosen from all 216 and 347 significant enriched GO terms, respectively. For large white pigs, these terms were mainly involved in immune, development of olfaction, and nervous process. As two of the most rapidly evolving genes, immune and olfaction‐related genes have been reported in many studies (Paudel et al., 2013, 2015). Two most significant terms of DU were “sensory perception” and “nervous system process,” showing a striking consistence with the result of DLW.

The top three enriched terms of MGI in DLW (Figure 3b) were the same as the parallel selection of DLW and DU (Figure S3b), which only had different orders. In addition, both of the fourth terms in DLW and DU (Figure 3b,d) were “Embryo,” which was mainly related to the development of embryo. Besides, we found that “Embryo” was significantly enriched in all of parallel, and DLW‐ and DU‐specific selections (Figure 3b,d and Figure S3b). In our study, “Mortality/Aging” was mainly related to embryonic lethality which may be caused by the abnormal development of embryo. *POLR1B* (RNA polymerase I subunit B), for example, can encode the second largest core subunit of RNA polymerase I (Pol I) which is essential for cell growth and inextricably linked to cell division (Russell & Zomerdijk, 2006), and it has been reported in recent studies to be associated with embryonic lethality (Chen et al., 2008; Derks et al., 2019). And these genes may play important roles during the strong artificial selection for litter size in recent years.

The results of GO and MGI showed striking consistence between parallel selection and population‐specific selection, which might result from the polygenic basis of quantitative traits. As the terms we showed such as nervous system, sensory perception, and growth are all quantitative traits, the minor genes of these traits may be under different selective pressures in the adaptive evolution of different pig breeds.

### 3.6 Population‐specific selection signatures also reveal the difference in selection for different purpose

Besides the polygenic basis of parallel selection traits, genes in population‐specific selection regions can also show some population‐specific traits. As shown in Table S11, QTLs identified in DLW and DU showed different enrichment levels in different traits. More specifically, QTLs related to “Health” and “Reproduction” are enriched in DLW, such as “CD8‐negative leukocyte percentage,” “teat number,” and “number of stillborn” (Table S9). In contrast, for DU, “Meat and Carcass”‐related QTLs such as “ham weight” and “marbling” were highly enriched (Table S10).

In the result of functional annotations, different trait‐related terms were enriched in DLW and DU. In consistent with QTLs, the GO terms related to immune such as “response to virus” were enriched in DLW and some growth‐related traits such as “limb development” and “skeletal system development” were enriched in DU (Figure 3a). By annotating with MGI database, both of DLW and DU were enriched with reproduction‐related term “Reproductive system,” and “Skeleton” had the highest enrichment level in DU which was in accordance with the results of GO annotation (Figure 3b,d). The enriched term “Reproductive system” in DU may be caused by the sperm‐related traits which were selected by better sire performance.

These results showed a significant difference between DLW and DU, which might be caused by their different breeding purposes. As dams and sires in pig industry, respectively, DLW was usually selected for high fertility, while DU was selected for growth and meat quality.

### 3.7 A series of potential causal variants played an important role in the process of artificial selection

**FIGURE 4:**
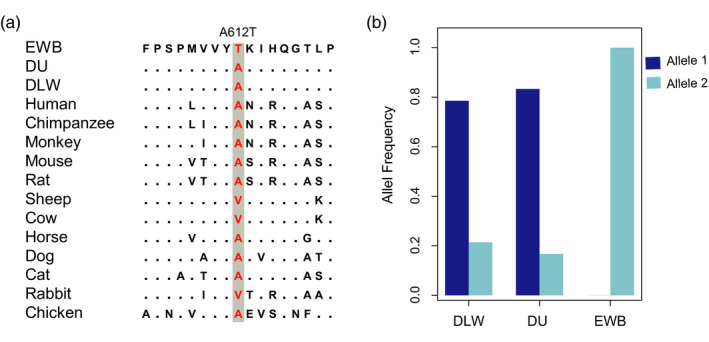
Putative causal variant (p. A612T) of parallel selection in CLCA1 gene. (c) Multispecies alignment of the protein sequences around the variant. The dots in the alignment indicate the amino acids which are identical with those in EWB, and the dashes indicate missing data. (d) Distribution of the allele frequency of the variant in three populations (DLW, DU, and EWB). DLW, Danish large white; DU, Duroc; EWB, European wild boar

For better interpreting the genetic basis under intense artificial selection, we thus annotated the SNPs within selection regions. In total, 19 nonsynonymous variants of seven genes were found with high absolute allele frequency difference (ΔAF > 0.8; Table S16) in parallel selection regions. As an example, the mutation (p. L192P) in *NR6A1* was also detected in previous study (Ribani et al., 2019) and is considered as a causative mutation which might influence the number of vertebrae (Mikawa et al., 2007). Interestingly, eight of these 19 variants were located in *CLCA2*, in spite of all these variants were predicted as tolerated alterations, indicating a selection that not be detected in our study such as the change in regulation region. In *CLCA1*, we found three such nonsynonymous variants, and one (p. A612T) of which was predicted as functional‐altering variant. This variant (p. A612T) showed a great difference between DLW, DU, and EWB (Figure 4).

We also noted that a total of 90 and 43 nonsynonymous variants with high absolute allele frequency difference (ΔAF > 0.8) were identified in large white (Table S17) and Duroc (Table S18), respectively, of which 10 and five variants were predicted as functional‐altering variants by SIFT. For large white‐specific selection, five of these 10 variants within four genes were highly conserved among multiple vertebrate species (Figure S4a–d). These genes (*HACE1*, *COL12A1*, *RNF111,* and *TLR10*) play important roles in many functions such as growth (Duester, 2008), movement (Punetha et al., 2017; Zou et al., 2014), embryonic development (Episkopou et al., 2001), and immunity (Cho et al., 2015; Stappers et al., 2015; Tang et al., 2015; Torices et al., 2016). For Duroc‐specific selection, one variant in *OCA2* gene was highly conserved. *OCA2* encodes the P protein, which is involved in mammalian pigmentation (Brilliant, 2001). And the candidate functional variant (p. R573H) which is located in a conserved region has also been reported to be associated with the pigmentation in pigs (Fernandez, Silio, Rodriguez, & Ovilo, 2006) (Figure S4e), indicating the identified variants can be served as functional variants in corresponding traits.

## 4 DISCUSSION

### 4.1 Complexity of parallel selection

Livestock such as pigs and sheep have undergone a long‐term artificial selection and many traits such as growth and fertility have been selected to meet human needs (Amaral et al., 2011; Chessa et al., 2009). As one of the genomic selection types, parallel selection exists widely in animals during the long‐term selection because of the similar needs for meat, egg, and milk traits in livestock farming (Colosimo et al., 2005; Frantz et al., 2015; Lamichhaney et al., 2017). As we know, most of the economic traits are complicated and generally controlled by many genes (called minor gene), while different breeds (such as large white and Duroc) under similar selective pressure, such as the selection for growth rate, different minor genes contributed to growth rate will be differently selected. In pig breeding history, different commercial populations have been bred for improving meat production through the genetic improvement in fertility, growth rate, and other economic traits. Although there are many parallel selected genes, we also found many genes were selected uniquely in only one breed. However, further bioinformatic analysis indicated that the signatures of parallel selection and population‐specific selection shared terms in the same traits. We interpret this phenomenon as a polygenic basis of parallel selection traits.

Under long‐term intense artificial selection, both of large white and Duroc pigs have shown superior performance in many traits such as growth rate. Besides the similar traits, many other traits were differently selected because of their different usage in industry. Large white pigs are usually used as dams, and for this purpose, the traits related to maternity and fertility are usually selected. In contrast, Duroc pigs are usually used as sires; thus, they are generally selected for growth rate and meat quality. But in the result of MGI analysis of parallel selection, we found that the term “reproduction system” was presented. This may be caused by the selection for sperm quality‐related traits of Duroc pigs. Sperm quality is usually used to measure the fertility of boars and indicates the sire effects in industry. In pig production, sperm quality such as sperm motility and concentration is usually evaluated to ensure the quality of sperm during the artificial insemination (AI) practice. By selecting the sperm quality, the genes involving in the fertility of Duroc pigs are selected, and thus shown in the “reproduction system” term.

### 4.2 Population‐specific selective signatures also implied different breeding objectives of large white and Duroc pigs

Although there are some shared terms of GO analyses of population‐specific selection, some terms were only enriched in large white or Duroc. For large white, the immune‐related terms were significantly enriched but not shown in Duroc. For different pig breeds, the immune‐related traits can have a different performance (Clapperton, Bishop, & Glass, 2005; Sutherland, Rodriguez‐Zas, Ellis, & Salak‐Johnson, 2005), especially for large white and Duroc pigs, the different living environments caused by different usage can lead to different selection for immune‐related traits. For Duroc, some terms about limb development were uniquely enriched such as “limb morphogenesis” and “appendage morphogenesis.” The limb development‐related terms may result from the strong selection on body size. This was consistent with the result of MGI annotation in DU which had the most significant term about “skeleton” (Figure 3d). The unique terms of large white and Duroc can be closely related to their population‐specific characters, and for different pig breeds, there must be wide selection signatures corresponding to the different characters remained for further studies. The same view could be drawn from the QTLs analysis. For DLW, “Health”‐related QTLs had the largest amount, which might reflect the rapid evolution of immune system. For DU, the most enriched QTL catalog was “Meat and Carcass,” which was the same as parallel selection, accounting for 83% of the total QTLs, and the main terms in “Meat and Carcass” were fat‐related terms.

## 5 CONCLUSIONS

In summary, we performed whole‐genome sequencing and genome‐wide scan of parallel selection in different pig breeds (large white and Duroc). Our analysis provided a general view of the parallel selection in large white and Duroc, and identified a series of relevant genes that are related to immunity, fertility, growth, and other functions. By summarizing the functional annotation of artificial selection, we suggested that parallel selection can be based on many genes for qualitative traits because of the polygenic basis. Moreover, we also identified six potential causative mutations of five genes involving multiple traits. Our results can advance our understanding of parallel selective process during intense artificial selection and provide multiple candidate genes and mutations which may contribute to the genetic breeding process.

## CONFLICT OF INTEREST

The authors declare that they have no conflict of interests regarding this publication.

## Supporting information

Supplementary Material

Table S11‐S18

## Data Availability

The data are all contained within the paper and Supporting Information files. Contact Yunlong.Ma@mail.hzau.edu.cn and xyli@mail.hzau.edu.cn for additional information. The data that we sequenced are available at Sequence Read Archive (SRP158574 and PRJNA‐658902). The other downloaded data for this study are available at SAMEA1557407, SAMEA1557419, SAMEA1557434, SAMN03031126, SAMN03031127, SAMN03031128, SAMN03031129, SAMN03031130, SAMN03031131, SAMN03031132, SAMN03031133, SAMN03031134, SAMN03031135, SAMN03031136, SAMN03031137, SAMN03031138, SAMN03031139, SAMN03031140, SAMN03031141, SAMN03031142, SAMN03031143, SAMN03031144, SAMN03031145, SAMEA1557387, SAMEA1557401, SAMEA1557403, SAMEA1557424, SAMEA1557433, SAMEA2612513, SAMEA2612514, SAMEA2612515, SAMEA2612516, SAMEA2612517, SAMEA2612518, SAMEA2612522, SAMEA2612523, SAMEA2612524, SAMEA2612525, SAMEA2612526; SAMEA3497874, SAMEA3497878, SAMEA3497880, SAMEA3497881, SAMEA3497882, SAMEA3497883, and SAMN02904855.
