# Supplementary material for: Selective sweep analysis reveals extensive parallel selection traits between large white and Duroc pigs
Source: Evol Appl. 2020 Aug 28;13(10):2807–20. doi: 10.1111/eva.13085 (PMC7691457; doi:10.1111/eva.13085)
Supplement: Supplementary file 1 — Supplementary Material [file EVA-13-2807-s001.docx]

**Selective sweep analysis reveals extensive parallel selection traits between Large white and Duroc Pigs**

Saixian Zhang, Kaili Zhang, Xia Peng, Huiwen Zhan, Jiahui Lu, Shengsong Xie, Shuhong Zhao, Xinyun Li^*^ and Yunlong Ma^*^

Key Laboratory of Agricultural Animal Genetics, Breeding, and Reproduction of the Ministry of Education & Key Laboratory of Swine Genetics and Breeding of the Ministry of Agriculture, Huazhong Agricultural University, Wuhan 430070, P. R. China

*Correspondence and requests for materials should be addressed to: Yunlong.Ma@mail.hzau.edu.cn and xyli@mail.hzau.edu.cn


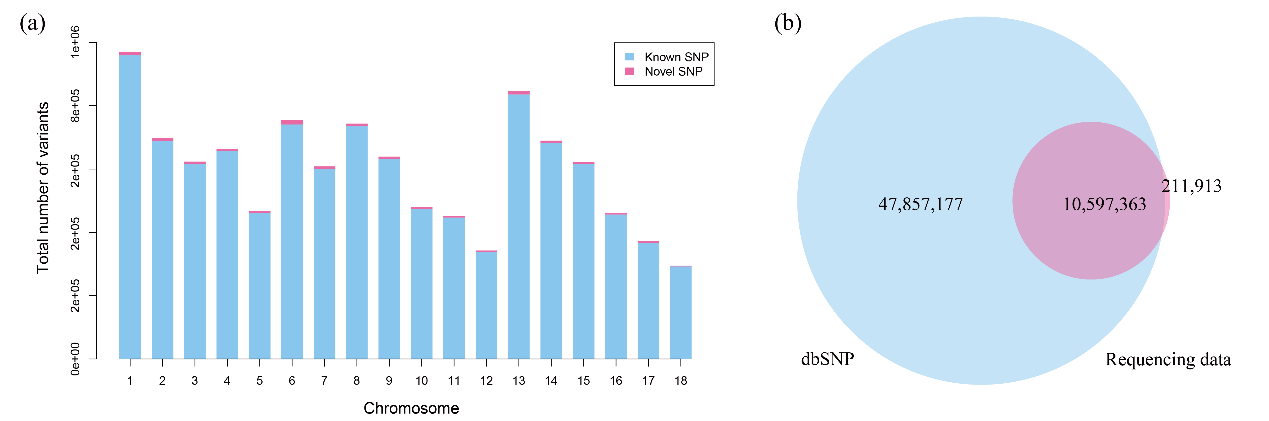


**Figure S1.** Statistic of SNPs and dbSNP dataset. (a) Distribution of SNPs in different chromosomes. “Known SNP” indicates the SNPs that have shown in dbSNP dataset, “Novel SNP” indicates the SNPs not shown in dbSNP dataset. (b) Venn plot of detected SNPs and dbSNP dataset. Pie colored with blue represents SNPs of dbSNP database, red color represent SNPs detected in this study.


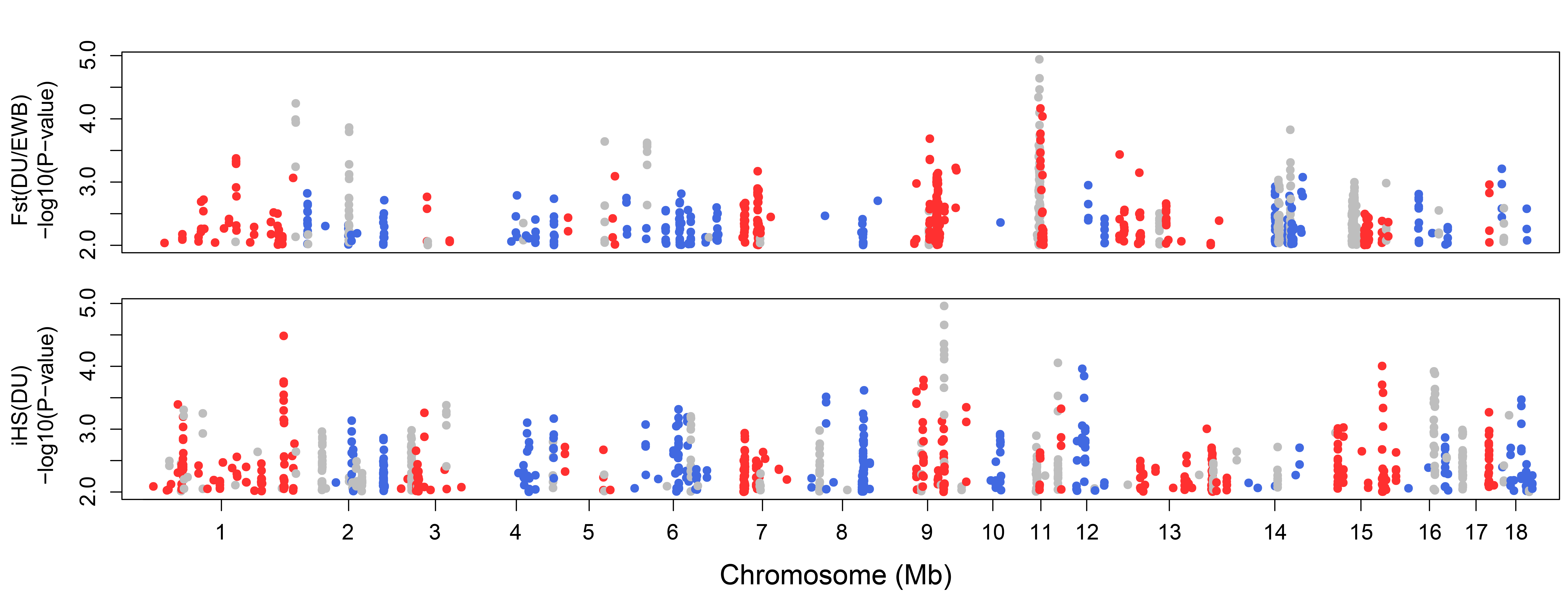


**Figure S2.** Candidate selection regions of DU detected by two statistics (Fst and iHS) are plotted across the genome. Red and blue dots represent the regions that are not identified as outliers in EWB with iHS method, gray dots represent the regions that are identified as outliers in EWB with iHS method.

**
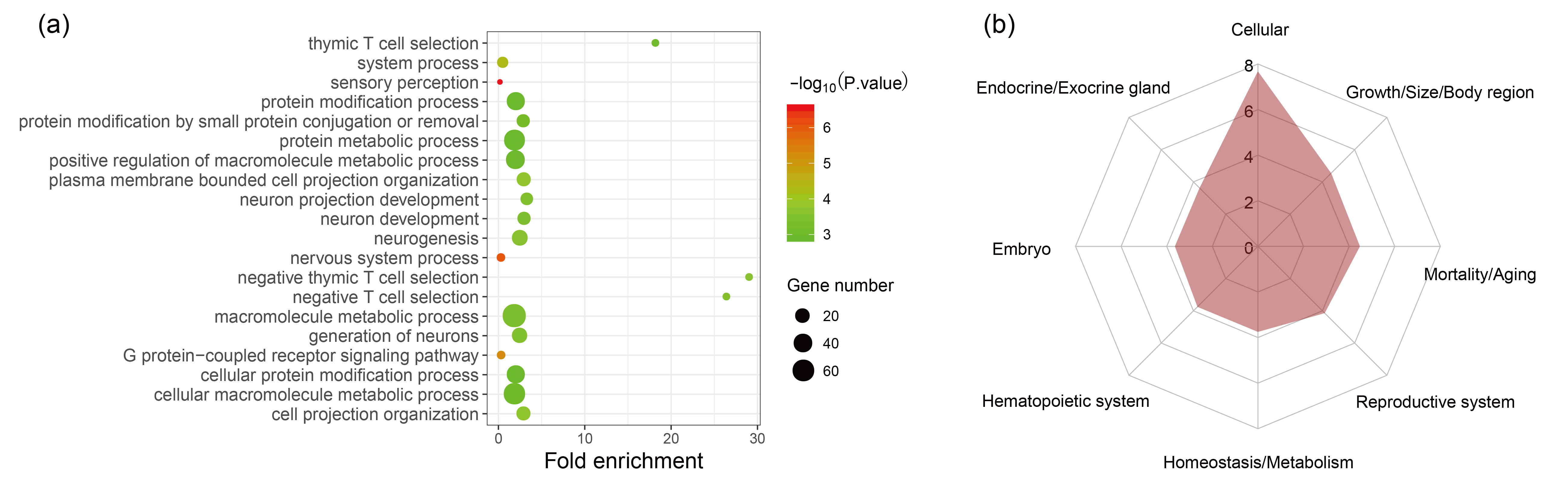
**

**Figure S3.** Annotation of the genes detected by parallel selection with GO and MGI databases. (a) Significantly enriched GO terms (top 20). (b) Significantly enriched MGI terms (top 8). The Y-axis in MGI plot indicates −log10(p-value).





**Figure S4.** Multispecies alignment of the protein sequences around putative causal variants. (a) p.L334V and p.S546N in TLR10 gene. (b) p.P728L in HACE1 gene. (c) p.S2543T in COL12A1 gene. (d) p.A625V in RNF111 gene. (e) p.R573H in OCA2 gene.

**Table S1.** Statistic of whole-genome sequencing data.

(see supplementary excel.)

**Table S2.** Significantly enriched GO terms of the 1,000 genes which contain the highest number of nonsynonymous variants.

(see supplementary excel.)

**Table S3.** Statistic of selective regions in three populations (DLW, DU and EWB) using two methods (Fst and iHS).

| Method | Items | Outliers regions | Candidate selection regions | Total length (Mb) | SNP counts |
| --- | --- | --- | --- | --- | --- |
| Fst | DLW/DU | 862 | 155 | 107.7 | 377,134 |
|  | DLW/EWB | 869 | 171 | 114.65 | 459,099 |
|  | DU/EWB | 869 | 147 | 113.55 | 447,636 |
| iHS | DLW | 906 | 353 | 195.8 | 882,725 |
|  | DU | 905 | 201 | 132.9 | 642,871 |
|  | EWB | 905 | 375 | 200.85 | 969,662 |

**Table S4.** Selective signatures of DLW and DU.

|  | Candidate selection regions | Total length (Mb) | Candidate genes counts | SNPs counts |
| --- | --- | --- | --- | --- |
| DLW | 392 | 227.20 | 2565 | 970326 |
| DU | 232 | 167.28 | 1660 | 749346 |

**Table S5.** Significantly enriched GO terms (top 20) of the genes in selection regions of DLW.

| GO Category | Terms | Gene counts | Fold Enrichment | P-value |
| --- | --- | --- | --- | --- |
| GO:0050907 | detection of chemical stimulus involved in sensory perception | 105 | 0.64 | 1.29E-06 |
| GO:0050911 | detection of chemical stimulus involved in sensory perception of smell | 105 | 0.65 | 2.25E-06 |
| GO:0007608 | sensory perception of smell | 107 | 0.65 | 2.59E-06 |
| GO:0007606 | sensory perception of chemical stimulus | 111 | 0.66 | 3.37E-06 |
| GO:0009593 | detection of chemical stimulus | 109 | 0.66 | 3.53E-06 |
| GO:0050906 | detection of stimulus involved in sensory perception | 111 | 0.66 | 4.03E-06 |
| GO:0007186 | G protein-coupled receptor signaling pathway | 143 | 0.72 | 2.61E-05 |
| GO:0048706 | embryonic skeletal system development | 28 | 2.64 | 2.67E-05 |
| GO:0051606 | detection of stimulus | 121 | 0.69 | 2.79E-05 |
| GO:0043009 | chordate embryonic development | 86 | 1.54 | 2.99E-04 |
| GO:0009792 | embryo development ending in birth or egg hatching | 87 | 1.53 | 3.27E-04 |
| GO:0007600 | sensory perception | 145 | 0.75 | 3.37E-04 |
| GO:0051607 | defense response to virus | 27 | 2.23 | 4.40E-04 |
| GO:0009790 | embryo development | 116 | 1.4 | 9.93E-04 |
| GO:0050877 | nervous system process | 175 | 0.79 | 1.47E-03 |
| GO:0007275 | multicellular organism development | 365 | 1.18 | 1.82E-03 |
| GO:0051254 | positive regulation of RNA metabolic process | 157 | 1.3 | 1.91E-03 |
| GO:0048856 | anatomical structure development | 395 | 1.17 | 1.93E-03 |
| GO:0021772 | olfactory bulb development | 10 | 3.42 | 2.12E-03 |
| GO:0086003 | cardiac muscle cell contraction | 9 | 3.69 | 2.29E-03 |

*Complete list is shown in **Table S6**.

**Table S7.** Significantly enriched GO terms (top 20) of the genes in selection regions of DU.

| GO Category | Terms | Gene counts | Fold Enrichment | P-value |
| --- | --- | --- | --- | --- |
| GO:0009593 | detection of chemical stimulus | 19 | 0.18 | 3.19E-25 |
| GO:0051606 | detection of stimulus | 22 | 0.2 | 4.87E-25 |
| GO:0050907 | detection of chemical stimulus involved in sensory perception | 19 | 0.18 | 9.89E-25 |
| GO:0050911 | detection of chemical stimulus involved in sensory perception of smell | 19 | 0.18 | 2.05E-24 |
| GO:0050906 | detection of stimulus involved in sensory perception | 21 | 0.2 | 2.36E-24 |
| GO:0007608 | sensory perception of smell | 20 | 0.19 | 3.98E-24 |
| GO:0007606 | sensory perception of chemical stimulus | 23 | 0.21 | 4.78E-23 |
| GO:0007600 | sensory perception | 40 | 0.32 | 1.43E-18 |
| GO:0007186 | G protein-coupled receptor signaling pathway | 48 | 0.38 | 5.56E-16 |
| GO:0050877 | nervous system process | 61 | 0.43 | 1.80E-14 |
| GO:0003008 | system process | 93 | 0.57 | 1.44E-09 |
| GO:0051252 | regulation of RNA metabolic process | 224 | 1.37 | 3.66E-06 |
| GO:0019219 | regulation of nucleobase-containing compound metabolic process | 235 | 1.35 | 5.40E-06 |
| GO:0048856 | anatomical structure development | 282 | 1.3 | 6.89E-06 |
| GO:0032502 | developmental process | 291 | 1.29 | 9.89E-06 |
| GO:0007275 | multicellular organism development | 259 | 1.31 | 1.64E-05 |
| GO:0048731 | system development | 236 | 1.32 | 1.72E-05 |
| GO:0010468 | regulation of gene expression | 247 | 1.31 | 2.71E-05 |
| GO:0003002 | regionalization | 37 | 2.19 | 3.92E-05 |
| GO:0006355 | regulation of transcription, DNA-templated | 201 | 1.34 | 3.95E-05 |

*Complete list is shown in **Table S8**.

**Table S9.** QTLs overlapped with selection regions of DLW.

(see supplementary excel.)

**Table S10.** QTLs overlapped with selection regions of DU.

(see supplementary excel.)

**Table S11.** Statistic of QTLs overlapped with selection regions of PS, DLW and DU.

| Catalogs | PS | DLW.sp | DU.sp | QTL database |
| --- | --- | --- | --- | --- |
| Exterior | 8 | 28 | 4 | 1978 |
| Health | 2 | 286 | 32 | 5475 |
| Meat and Carcass | 16 | 198 | 205 | 10940 |
| Production | 0 | 27 | 4 | 1843 |
| Reproduction | 0 | 37 | 2 | 1716 |
| Total | 26 | 576 | 247 | 21952 |

PS: parallel selection of DLW and DU, DLW.sp: DLW-specific selection, DU.sp: DU-specific selection

**Table S12.** QTLs overlapped with regions of parallel selection.

(see supplementary excel.)

**Table S13.** Significantly enriched GO terms of the genes in parallel selection regions

(see supplementary excel.)

**Table S14.** QTLs overlapped with regions of DLW-specific selection.

(see supplementary excel.)

**Table S15.** QTLs overlapped with regions of DU-specific selection.

(see supplementary excel.)

**Table S16.** Candidate nonsynonymous variants (ΔAF > 0.8) of parallel selection.

(see supplementary excel.)

**Table S17.** Candidate nonsynonymous variants (ΔAF > 0.8) of DLW-specific selection.

(see supplementary excel.)

**Table S18.** Candidate nonsynonymous variants (ΔAF > 0.8) of DU-specific selection.

(see supplementary excel.)
